# Pharmacokinetics and pharmacodynamics of bioactive compounds in Penyanqing preparation in THP-1 inflammatory cells induced by Lipopolysaccharide

**DOI:** 10.1186/s12906-022-03784-x

**Published:** 2022-12-06

**Authors:** Linna Gong, Zhishuo Miao, Li Zhang, Birui Shi, Zuoqi Xiao, Panzi Qiu, Menghua Liu, Wei Zou

**Affiliations:** 1 Biopharmaceutics, NMPA Key Laboratory for Research and Evaluation of Drug Metabolism, School of Pharmaceutical Sciences, Southern Medical University, Guangzhou, 510515 China; 2 Changsha Research and Development Center On Obstetric and Gynecologic Traditional Chinese Medicine Preparation, NHC Key Laboratory of Birth Defects Research, Prevention and Treatment, Hunan Provincial Maternal and Child Health Care Hospital, Changsha, 410008 Hunan China

**Keywords:** Penyanqing preparation, Pelvic inflammatory disease, Pharmacokinetics, THP-1 macrophage cells, HPLC–MS/MS

## Abstract

**Background:**

Penyanqing (PYQ), a traditional Chinese medicine (TCM), has a good clinical efficacy for the treatment of pelvic inflammatory disease (PID). Previously, researches on its anti-inflammatory effect and mechanism in vitro*, *in silico*,* and in vivo have been reported by our team. However, the interrelationship between the anti-inflammatory activity and the active compounds in PYQ are not clear. Here, the pharmacokinetics-pharmacodynamics (PK-PD) study was carried out for more proper clinical use.

**Methods:**

The plasma concentrations of salvianolic acid B (SAB), protocatechualdehyde (PRO), paeoniflorin (PE), astilbin (AST), ferulic acid (FE), and chlorogenic acid (CH) in SD rats after PYQ administration were determined by a selective and rapid HPLC–MS/MS method. In addition, the PK-PD on cell model was used to explore the relationship between the plasma concentration and inflammatory biomarkers (TNF-α, IL-1β).

**Results:**

The results of this study showed that the six components could reach the peak blood concentration within 0.29 h, indicating the rapid absorption of it. The eliminations of AST, CH, FE, PE, and PRO were relatively fast due to their mean residence times (MRTs) within 3 h, while the elimination of SAB was slower (MRT 5.67 ± 0.66 h). Combined with a THP-1 cell model, there was a significant correlation between inflammatory factors and component plasma concentrations with correlation coefficients in the range of -0.9—-0.746. Correspondingly, the drug-containing plasma obtained at 0.25 h point exhibited the best inhibition effect on production of IL-1β and TNF-α in LPS-induced THP-1 cells.

**Conclusion:**

The six main components in PYQ could be quickly absorbed, and there was a potential good correlation between their pharmacokinetics and the pharmacodynamics of PYQ.

**Supplementary Information:**

The online version contains supplementary material available at 10.1186/s12906-022-03784-x.

## Introduction

Pelvic inflammatory disease (PID) is a common gynecological disease, which may lead to a variety of complications such as infertility, ectopic pregnancy, and chronic pelvic pain [1]. Currently, antibiotics are the first choice in the treatment, while they are not effective in the treatment of chronic inflammation and sequelae of PID, and long-term use may lead to drug resistance. Fortunately, clinical effect proves that traditional Chinese medicine (TCM) has made up for the deficiency of antibiotics in the treatment of PID [2]. Penyanqing (PYQ) is prepared by the Hunan Provincial Maternal and Child Health Care Hospital with eleven Chinese medicinal materials (Table 1) [3]. In clinic, PYQ has good curative effects on chronic PID, including the relief of pain caused by PID, inhibition of thrombosis, and improvement the body’s immune function [4]. Further, we have carried out a series of investigation on the anti-inflammatory mechanism of PYQ [5–7]. It was confirmed that PYQ could suppress the infiltrations of lymphocytes and neutrophils in the uterine tube, decrease the release of interleukin IL-1β, IL-6, IL-8, monocyte chemotactic protein (MCP)-1, and promote the production of lipoxin A_4_ (LXA_4_). On the other hand, PYQ regulated the activity of NF-κB signal pathway and promoted the expression of FPR2 on the LPS-stimulated THP-1 cell line, which suggested the potential mechanisms of its anti-inflammatory effect [6]. In addition, we performed molecular docking analysis and network analysis to screen significant effective compounds and key targets. Six active compounds, including paeoniflorin (PE), salvianolic acid B (SAB), protocatechualdehyde (PRO), astilbin (AST), ferulic acid (FE), and chlorogenic acid (CH) were found. The GO (Gene Ontology) and KEGG (Kyoto encyclopedia of genes and genomes) pathway enrichment analysis indicated that PYQ exerted anti-inflammatory effects mainly through several pathways, such as IRAK1-NF-κB pathway, Toll-like receptor signaling pathway, and NOD-like receptor signaling pathway [7]. However, the relationship between the anti-inflammatory effect and the main absorbable compounds is unclear.

**Table 1:** Eleven Chinese medicinal materials in PYQ

Chinese name	Latin name
Danshen	*Salvia miltiorrhiza* Bge
Danggui	*Angelica sinensis (*Oliv.) Diels
Chishao	*Paeonia lactiflora* Pall
Yanhusuo	*Corydalis yanhusuo* W.T.Wang
Baijiangcao	*Patrinia scabiosaefolia* Fisch. ex Trev
Xiangfu	*Cyperus rotundus* L
Sanleng	*Sparganium stoloniferum* Buch.-Ham
Wuyao	*Lindera aggregate* (Sims) Kos-term
Gancao	*Glycyrrhiza uralensis* Fisch
Tufuling	*Smilax glabra* Roxb
Daxueteng	*Sargentodoxa cuneata (*Oliv.) Rehd. et Wils

It is reported that the FE in *Angelica sinensis (Oliv.)* Diels has pharmacological effects such as antiplatelet aggregation, antithrombotic, antibacterial, anti-inflammatory, and enhancing immune function [8]. SAB in *Salvia miltiorrhiza* Bge. is the main effective component with antiplatelet aggregation, antithrombotic, and anti-inflammatory properties [9, 10]. PE is the main effective component of *Paeonia lactiflora* Pall. in reducing blood viscosity, antiplatelet aggregation, antithrombus, dilating blood vessels, improving microcirculation, and anti-inflammation [11, 12]. Pharmacological studies show that CH is an important anticoagulant and antibacterial component of *Angelica sinensis (Oliv.)* Diels [13]. These researches supported our previous study that these components may be the main active components of PYQ for its anti-inflammatory effect in the treatment of PID [6]. Moreover, SAB, PE, and FE have been used as an index component for the quality control of *Salvia miltiorrhiza* Bge., *Paeonia lactiflora* Pall., and *Angelica sinensis (Oliv.)* Diels in Chinese Pharmacopoeia, respectively [14–18]. Pharmacokinetic research of TCM compound is particularly necessary because it could illuminate the synergistic effect of multiple active components to exert the pharmacological efficacy [19–23]. Therefore, in the present paper, a sensitive and reliable HPLC–MS/MS method was established for simultaneous determination of SAB, PRO, PE, AST, FE, and CH in rat plasma, and the pharmacokinetics- pharmacodynamics (PK-PD) study on cell model was used to explore the relationship between the plasma concentrations and inflammatory biomarkers (TNF-α, IL-1β), so as to providing a scientific basis for the clinical application of PYQ.

## Materials and methods

### Chemicals and reagents

**Fig. 1:**
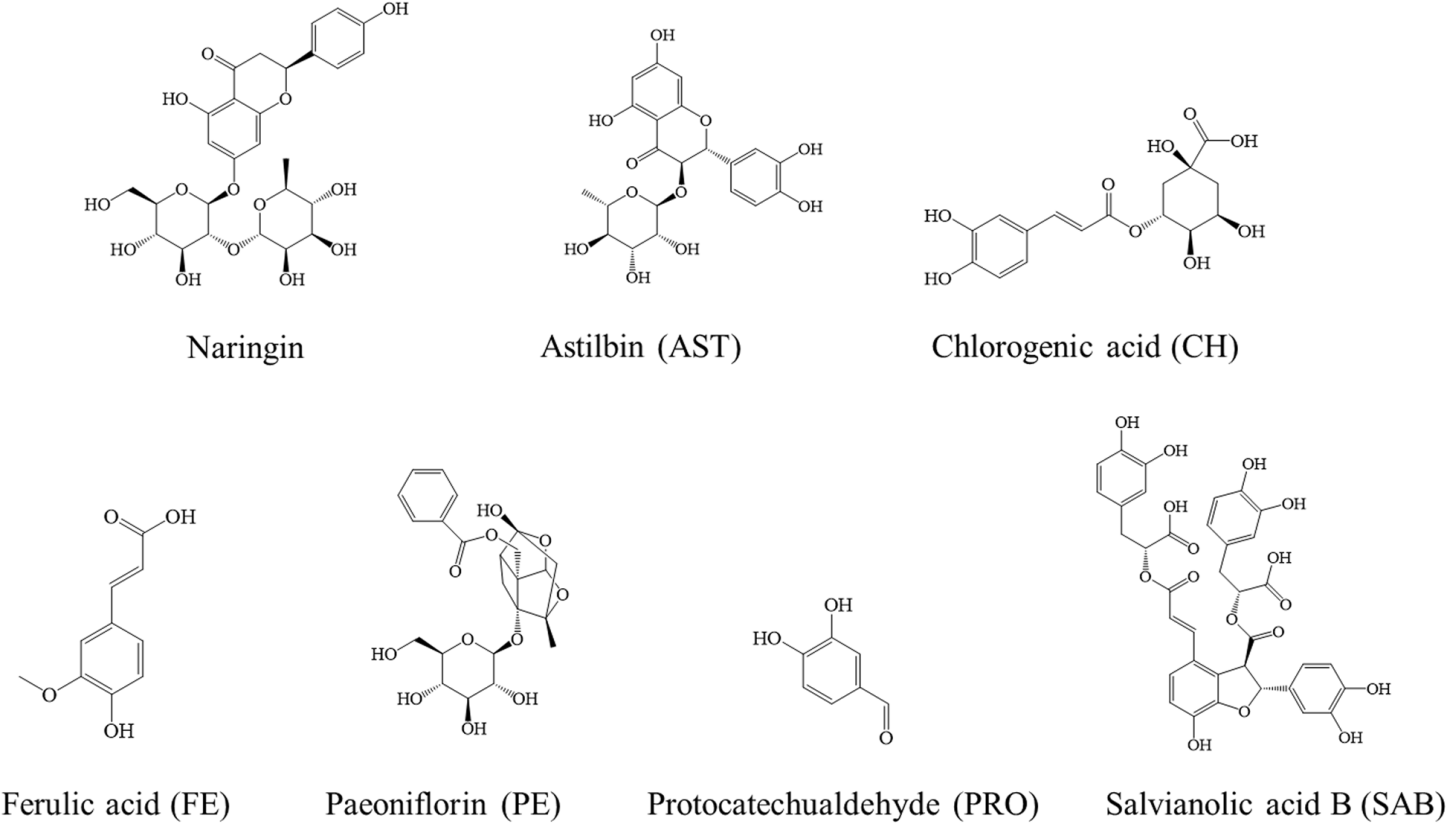
Chemical structures of the six candidate compounds and naringin

PYQ was obtained from the Hunan Provincial Maternal and Child Health Care Hospital, with batch numbers of 20,190,704. SAB, PRO, PE, AST, FE, CH, and naringin (IS) were purchased from the National Institutes for Food and Drug Control. Their chemical structures are presented in Fig. 1. Ethyl acetate was purchased from Shanghai Aladdin Biochemical Technology Co., Ltd. Methanol was chromatographic grade and obtained from Merck (Darmstadt, Germany). Formic acid solution and heparin sodium were purchased from Sigma Co. (St Louis, MO). Deionized water was purified by a Milli-Q Ultrapure water system (Millipore, Milford, MA, USA). All of the other reagents were analytical grade. The human IL-1β, and TNF-α ELISA kits were purchased from Beijing 4A Biotech Co., Ltd. Gibco RPMI-1640 Medium, Fetal Bovine Serum (FBS), and Penicillin–Streptomycin solution were purchased from Thermo Fisher Scientific Inc. (Waltham, MA, USA). Phorbol 12-myristate 13-acetate (PMA) was obtained from MedChemExpress. Lipopolysaccharide (LPS) was provided by Sigma-Aldrich. Insulin was purchased from Dalian Meilun Biotechnology Co., Ltd. The THP-1 cell lines were supplied by Shanghai Cell Bank, Chinese Academy of Sciences (Shanghai, China).

### Animal experimental

Animal experiments referred to our previous manuscript [6]. Female Sprague–Dawley rats (*n* = 10), weighing 200 ± 20 g, were purchased from the Experimental Animal Center of Southern Medical University (Guangzhou, China). These rats were kept adaptively for one week before the experiment, with a temperature of 23 ± 2 ℃, humidity of 40 ± 5%, 12 h light/dark cycle, and random-fed with water and food available. The rats were fasted overnight before administration.

In our previous study, three different doses (5, 10, and 20 mL/kg/day) of PYQ were used to explore the pharmacodynamic effects of it. According to the clinical dose, the PYQ was orally administered to rats at a dose of 8 mL/kg in in this experiment. The plasma of 500 µL was obtained via the retinal venous plexus at 0.083, 0.25, 0.5, 0.75, 1, 2, 4, 8, 12 and 24 h after gavage administration. Then all samples were centrifuged at 4 ℃ for 5 min at 8,000 rpm, and were stored at -80 ℃ in refrigerator until further analysis.

### Plasma sample preparation

#### Plasma sample preparation of PRO, PE, AST, FE, and CH

For the pharmacokinetic study of PRO, PE, AST, FE, and CH, 100 µL plasma sample was mixed with 10 µL internal standard (IS, 2 µg/mL naringin), and 700 µL methanol was added for protein sedimentation. The mixture was vortexed for 6 min and centrifuged at 13, 000 rpm for 15 min at 4 ℃. Next, the supernatant was dried in a vacuum drying oven and vortexed with 100 µL of 50% methanol for 6 min, and then the mixture was centrifuged at 13, 000 rpm for 30 min at 4 ℃, and the supernatant was filtered by 0.22 µM filter membrane for HPLC–MS/MS analysis.

#### Plasma sample preparation of SAB

For the pharmacokinetic study of SAB, 100 µL plasma was mixed with 10 µL IS (2 µg/mL naringin) and then acidified by mixing with 25 µL of 20% hydrochloric acid. After addition of 1 mL of ethyl acetate, the mixture was vortexed for 6 min and centrifuged at 13,000 rpm for 15 min. The organic layer was transferred to an Eppendorf tube and dried in a vacuum drying oven. The residue was dissolved in 100 µL methanol. After centrifugation at 13,000 rpm for 30 min, the supernatant was filtered by 0.22 µM filter membrane for HPLC–MS/MS analysis.

#### LC–MS/MS method for pharmacokinetics study

In this study, the negative ESI multiple reaction monitoring (MRM) mode of AB Sciex API-4000 LC–MS/MS Systems was used to measure SAB, PRO, PE, AST, FE, and CH in rat plasma, respectively. The following setups of the analyzers were used: the temperatures were all 500 ℃; GS1, 55 psi; GS2, 55 psi; curtain gas, 10 psi. The mass spectrum parameters of 6 ingredients and IS in rat plasma were shown in Table 2.

**Table 2:** Mass spectrum parameters of 6 analytes and internal standard (IS) in rat plasma

Compound	Molecular weight	Parent ion	Fragment ion
AST	450.408	449.3	151.1
FE	194.19	193.0	177.6
PRO	138.122	136.7	107.4
CH	354.31	353.1	190.8
PE	480.45	479.0	448.9
SAB	718.62	717.4	519.4
IS	580.53	579.1	271.0

For the chromatographic analysis of PRO, PE, AST, FE, and CH, the chromatographic column used was Agilent Poroshell 120 EC-C18 (100 mm × 3.0 mm, 2.7 µm), and the column temperature was maintained at 25 ℃. The mobile phase was consisted of A (methanol) and B (0.2% aqueous formic acid), and the flow rate was 0.4 mL/min. The gradient elution was carried out as follows: 10% A at 0 ~ 1 min, 10% A-95% A at 1 ~ 2 min, 95% A at 2 ~ 5 min, 95% A-10% A at 5 ~ 5.1 min, 10% A at 5.1 ~ 6 min. 5 µL sample was injected into the system for analysis.

For the chromatographic analysis of SAB, the chromatographic column used was ZORBAX SB-C18 (100 mm × 2.1 mm, 3.5 µm), and the column temperature was maintained at 25 ℃. The mobile phase was consisted of A (methanol) and B (0.2% aqueous formic acid), and the flow rate was 0.4 mL/min. The gradient elution was carried out as follows: 30% A at 0 ~ 0.5 min, 30% A-90% A at 0.5 ~ 1 min, 90% A at 1 ~ 4.5 min, 90% A-30% A at 4.5 ~ 4.6 min, 30% A at 4.6 ~ 6 min. 5 µL sample was injected into the system for analysis.

### Preparation of calibration standards and quality control (QC) samples

Stock solutions of SAB, PRO, PE, AST, FE, CH, and IS were prepared in methanol. Stock solutions of these 6 compounds were serially diluted with methanol to provide suitable standard working solutions. The following calibration standards were generated: 50–600 ng/mL for AST, 25–500 ng/mL for FE, 10–500 ng/mL for PRO, 50–600 ng/mL for CH, 50–600 ng/mL for PE, 100–1000 ng/mL for SAB. 10 µL of each calibration standard was added to 100 µL of blank plasma respectively, and mixed by vortexing for 1 min. The mixtures were followed the same method of the treatment of plasma samples to obtain the standard calibration samples.

QC samples were prepared as the same method of the standard calibration samples at 10, 20, 500 ng/mL for AST, 50, 125, 400 ng/mL for FE, 20, 100, 400 ng/mL for PRO, 100, 200, 500 ng/mL for CH, 100, 200, 500 ng/mL for PE, 200, 400, 800 ng/mL for SAB respectively. All the stock solutions, working solutions and QC samples were stored at 4 ℃ until use.

### Bioanalytical method validation

The specificity of the method was determined by comparing chromatograms of blank plasma collected from ten different rats, blank plasma spiked with the mixed standards and IS, and plasma samples obtained after oral administration of PYQ.

Calibration curve was prepared by spiking pooled blank matrix with 6 calibration standards. Calibration curve was calculated using weighted linear least squares regression model by plotting peak area ratio of each analyte to IS versus concentrations of the analytes. The lower limit of quantification was considered as the lowest level of calibration curve with precision and accuracy lower than 20%.

The precision and accuracy of the method were determined by performing 6 replicates of QC samples spiked with three concentration levels (low, middle and high) on the same day (intra-day) and on 3 consecutive days (inter-day). The precision and accuracy of analytical procedure at each QC concentration were expressed as the RSD and RE.

The extraction recovery was determined by comparing the peak area for postextraction blank plasma-spiked compounds against preextraction blank plasma added compounds at three QC levels. The matrix effects were determined by comparing the peak responses of compounds spiked into the pretreated blank rat plasma with those of corresponding concentration pure standard solutions, the IS was determined through a similar process method.

The stability tests of the analytes were evaluated by analyzing six replicates of QC samples at three levels under different storage conditions: 24 h at room temperature (about 25 ℃), three freeze–thaw cycles, and incubated at 56 ℃ for 1 h. The stability of all QC samples was determined by the freshly calibration curve.

### PK-PD study in THP-1 Cells

THP-1 cells were cultivated in RPMI-1640 Medium with 10% FBS and 1% antibiotic solution of Penicillin–Streptomycin solution. THP-1 cells were divided into control group, blank plasma group and PYQ drug-containing group with different pharmacokinetic plasma collection time points (0.083, 0.25, 0.5, 0.75, 1, 2, 4, 8, 12, 24 h). PMA with a concentration of 100 ng/mL was added to THP-1 cells at a density of 2 × 10^5^ cells/mL in 12-well culture plates and maintained at 5% CO_2_, 37 ℃ for 48 h. The cell through PMA-induced differentiation displayed a macrophage morphology and adhered to the culture plate. We determined the effects of PYQ and its active compounds on THP-1 cell viability after 24 h of treatment in the preliminary experiments and chose a drug concentration that had no effect on cell viability as the drug concentration for THP-1 cells (Figure S1). According to the reference [24, 25], 1.8 mL of drug-containing plasma and blank plasma (15%) inactivated in 56 ℃ water bath for 30 min and filtered by 0.22 µm microporous membrane at different pharmacokinetic plasma collection time points were added to 12 mL of culture medium. After 2 h of culture, LPS solution was added to each group except the control group to make the final concentration reach 1 µg/mL, and the culture was continued for 24 h in a 37 ℃, 5% CO_2_ incubator. After that, the cell culture solution of each group was collected, centrifuged at 1000 g for 10 min, and the supernatant was used to detect the levels of IL-1β, and TNF-α. The levels of IL-1β and TNF-α were tested by ELISA kit according to the manufacturer’s instructions.

### Data analyses

The pharmacokinetic parameters were calculated by non-compartmental model of the DAS 2.0 software package. The data were presented as mean ± standard deviation (SD). Statistical calculations were performed using SPSS statistical software, and a value of *p* < 0.05 was denoted statistically significant difference.

## Results

### Specificity

**Fig. 2:**
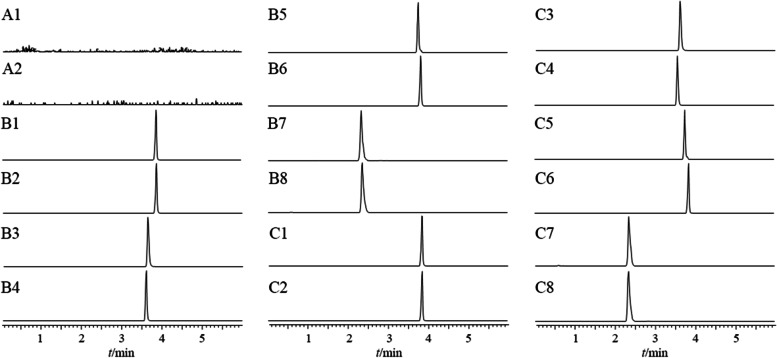
MRM chromatograms of 6 ingredients and internal standard. A1: MRM chromatograms of blank plasma for AST, FE, PRO, CH and PE; A2: MRM chromatograms of blank plasma for SAB. B1: MRM chromatograms of AST in blank plasma; B2: MRM chromatograms of FE in blank plasma; B3: MRM chromatograms of PRO in blank plasma; B4: MRM chromatograms of CH in blank plasma; B5: MRM chromatograms of PE in blank plasma; B6: MRM chromatograms of IS in blank plasma according to 2.3.1; B7: MRM chromatograms of SAB in blank plasma; B8: MRM chromatograms of IS in blank plasma according to 2.3.2; C1: MRM chromatograms of AST in plasma; C2: MRM chromatograms of FE in plasma; C3: MRM chromatograms of PRO in plasma; C4: MRM chromatograms of CH in plasma; C5: MRM chromatograms of PE in plasma; C6: MRM chromatograms of IS in plasma according to 2.3.1; C7: MRM chromatograms of SAB in plasma; C8: MRM chromatograms of IS in plasma according to 2.3.2

The chromatograms of blank rat plasma, blank plasma spiked with mixture of six compounds and IS, and the plasma samples collected after administration of PYQ are displayed in Fig. 2. There was no endogenous interference observed at the retention times of the analytes and IS. AST, FE, PRO, CH, PE, IS1, SAB, and IS2 have good peak shapes with retention times of 3.84 min, 3.86 min, 3.62 min, 3.58 min, 3.76 min, 3.81 min, 2.33 min, and 2.34 min, respectively.

### Calibration curve and lower limit of quantification (LLOQ)

The calibration curves of six compounds all showed good linearities, and the correlation coefficients were higher than 0.999. The accuracy and relative RSD of the LLOQ of each analyte meet the requirements of biological sample analysis method. Table 3 shows the standard curve, correlation coefficient, linear range and LLOQ of each compound in rat blank plasma.

**Table 3:** Regression equations, correlation coefficients, linear ranges, lower limit of quantification of 6 ingredients (*n* = 6)

Compounds	Regression equations	Correlation coefficients	Linear ranges (ng/mL)	LLOQ (ng/mL)
AST	Y = 0.0006 X + 0.0099	0.9992	50—600	50
FE	Y = 0.0033 X + 0.1772	0.9992	25—500	25
PRO	Y = 0.0028 X + 0.0346	0.9993	10—500	10
CH	Y = 0.0112 X—0.0449	0.9990	50—600	50
PE	Y = 0.0001 X—0.0002	0.9994	50—600	50
SAB	Y = 0.0006 X—0.0030	0.9993	100—1000	100

### Extraction recovery, matrix effect, precision, accuracy, and stability

As shown in Table 4, the extraction recoveries of analysts were in the range of 87.79%-97.99%. The matrix effects of analysts were ranged from 88.97% to 110.06%. The intra-day and inter-day precisions of analysts were ranged from 1.13% to 8.48% and from 1.84% to 6.33%, respectively, and accuracy was between 90.92% and 104.00%. The results demonstrated that the method had satisfactory precision and accuracy. The results of stability showed that all RSD were less than 5%, indicating that the analysts were stable after 24 h at room temperature, after undertaking three freeze–thaw cycles, and after 1 h at 56 ℃.

**Table 4:** The results of matrix effects, recovery, precision, accuracy and stability of six ingredients in plasma (*n* = 6)

Compounds	Concentration (ng/mL)	matrix effects (%)	Recovery (%)	Precision (RSD%)		Accuracy (%)		Room temperature 24 h	Three freeze- thaw cycles	In 56 ℃ for 1 h
				**Intra-day**	**Inter-day**	**Intra-day**	**Inter-day**	**measured value**	**measured value**	**measured value**
	100	96.55 ± 2.09	95.92 ± 2.07	5.85 ± 5.78	5.19 ± 5.18	98.73 ± 5.78	99.76 ± 5.18	99.68 ± 2.31	97.89 ± 4.06	98.46 ± 2.77
AST	200	102.99 ± 2.75	92.76 ± 2.47	2.85 ± 2.71	3.98 ± 3.87	95.05 ± 2.71	97.36 ± 3.87	199.01 ± 3.99	193.37 ± 7.61	194.85 ± 6.53
	500	100.24 ± 2.28	94.96 ± 2.16	2.86 ± 2.82	2.43 ± 2.43	98.93 ± 2.82	99.96 ± 2.43	498.07 ± 3.95	497.15 ± 6.61	495.21 ± 8.43
	50	107.61 ± 2.45	89.83 ± 2.04	7.89 ± 8.08	6.33 ± 6.35	102.43 ± 8.08	100.4 ± 6.35	49.14 ± 2.35	49.41 ± 1.85	48.33 ± 2.76
FE	125	93.48 ± 4.51	90.13 ± 4.35	2.18 ± 2.19	2.61 ± 2.63	100.37 ± 2.19	100.68 ± 2.63	122.72 ± 2.65	124.36 ± 2.46	123.42 ± 1.76
	400	88.56 ± 2.09	96.54 ± 2.74	1.45 ± 1.48	1.84 ± 1.88	102.23 ± 1.48	101.92 ± 1.88	403.59 ± 7.84	400.59 ± 6.95	399.13 ± 3.26
	20	92.25 ± 2.35	95.84 ± 2.44	8.48 ± 8.75	6.01 ± 6.25	103.23 ± 8.7	104 ± 6.25	20.81 ± 0.85	20.62 ± 1.06	19.88 ± 0.43
PRO	100	108.34 ± 1.53	90.61 ± 1.28	6.67 ± 6.62	4.78 ± 4.89	99.17 ± 6.62	102.15 ± 4.89	100.34 ± 1.48	97.77 ± 2.49	98.21 ± 2.33
	400	105.24 ± 3.06	91.32 ± 2.65	4.3 ± 4.43	3.45 ± 3.48	103 ± 4.43	101.05 ± 3.48	398.27 ± 4.21	395.66 ± 7.14	394.26 ± 8.01
	100	103.81 ± 3.10	89.57 ± 2.67	4.51 ± 4.36	3.62 ± 3.56	96.56 ± 4.36	98.34 ± 3.56	99.91 ± 2.94	98.55 ± 2.93	97.22 ± 3.31
CH	200	102.10 ± 2.23	95.61 ± 3.02	4.96 ± 4.69	5.13 ± 4.96	94.62 ± 4.69	96.84 ± 4.96	200.03 ± 9.56	191.77 ± 8.91	195.56 ± 7.88
	500	93.58 ± 1.13	97.97 ± 1.18	6.84 ± 6.75	4.57 ± 4.54	98.66 ± 6.75	99.58 ± 4.54	499.00 ± 6.02	485.90 ± 9.73	489.60 ± 8.91
	100	102.84 ± 2.17	90.62 ± 1.91	5.87 ± 5.68	4.09 ± 4.03	96.67 ± 5.68	98.7 ± 4.03	99.44 ± 2.43	99.68 ± 2.83	95.32 ± 1.06
PE	200	107.07 ± 0.71	98.69 ± 0.65	1.71 ± 1.75	2.53 ± 2.60	102.79 ± 1.75	102.68 ± 2.60	199.64 ± 2.59	197.55 ± 3.60	196.25 ± 4.08
	500	100.02 ± 6.36	94.55 ± 6.01	1.83 ± 1.84	2.6 ± 2.62	100.82 ± 1.84	100.71 ± 2.62	499.23 ± 2.58	492.85 ± 7.60	495.87 ± 6.50
	200	91.94 ± 3.62	97.90 ± 3.85	3.83 ± 3.69	5.12 ± 5.10	96.23 ± 3.69	99.64 ± 5.10	208.88 ± 8.46	194.10 ± 8.56	196.22 ± 3.40
SAB	400	101.21 ± 1.16	92.44 ± 1.06	1.13 ± 1.03	3.2 ± 2.95	90.92 ± 1.03	92.09 ± 2.95	391.06 ± 6.16	388.99 ± 6.75	395.20 ± 10.70
	800	102.70 ± 1.34	99.81 ± 1.30	3.57 ± 3.39	3.2 ± 2.97	94.98 ± 3.39	92.82 ± 2.97	792.27 ± 3.94	790.71 ± 3.52	791.11 ± 5.98

### Pharmacokinetic and pharmacodynamic application

**Fig. 3:**
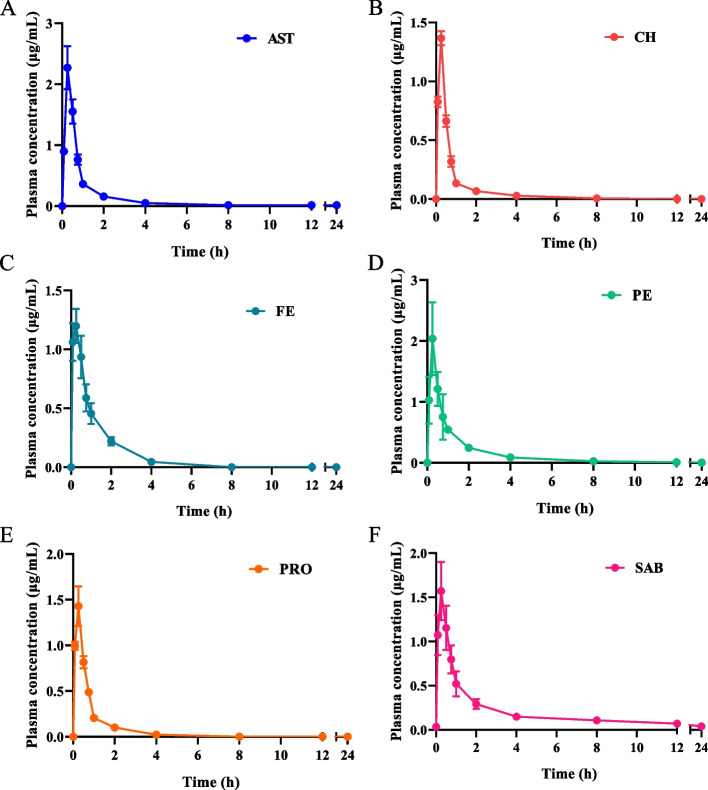
The plasma concentration–time curves of six compounds. Samples at 0 points were corresponded to blank plasma group. Samples at other time points were corresponded to plasma collected at 0.083, 0.25, 0.5, 0.75, 1, 2, 4, 8, 12, and 24 h, respectively (mean ± SD, *n* = 6)

The validated LC–MS/MS method was applied to simultaneous determination of the six compounds in plasma samples after oral administration of PYQ in rats. The mean plasma concentration–time curves of the six compounds were shown in Fig. 3. The pharmacokinetic parameters of these six compounds were summarized in Table 5. Results showed that the *t*_*max*_ of AST, CH, FE, PE, and PRO were 0.25 h, and the *t*_*max*_ of SAB was 0.29 h.

**Table 5:** Pharmacokinetics parameters for six compounds (mean ± SD, *n* = 6)

Parameter	Unit	AST	CH	FE	PE	PRO	SAB
*AUC*_*(0-t)*_	mg/L*h	2.08 ± 0.20	0.92 ± 0.06	1.43 ± 0.23	2.21 ± 0.31	1.05 ± 0.06	3.40 ± 0.45
*AUC*_*(0-∞)*_	mg/L*h	2.09 ± 0.20	0.94 ± 0.09	1.48 ± 0.24	2.22 ± 0.31	1.08 ± 0.07	3.70 ± 0.46
*t*_*1/2*_	h	3.42 ± 0.34	1.86 ± 0.21	0.90 ± 0.02	2.37 ± 0.20	0.93 ± 0.06	7.60 ± 3.34
*t*_*max*_	h	0.25 ± 0	0.25 ± 0	0.25 ± 0	0.25 ± 0	0.25 ± 0	0.29 ± 0.10
*CLz/F*	L/h/kg	9.55 ± 0.81	21.33 ± 1.29	13.80 ± 2.37	9.15 ± 1.27	18.57 ± 1.07	5.48 ± 0.68
*Vz/F*	L/kg	45.37 ± 6.31	57.19 ± 7.48	17.85 ± 3.28	31.26 ± 4.08	24.94 ± 1.96	59.83 ± 25.48
*C*_*max*_	mg/L	2.27 ± 0.35	1.37 ± 0.06	1.20 ± 0.15	2.04 ± 0.59	1.43 ± 0.22	1.59 ± 0.33

**Fig. 4:**
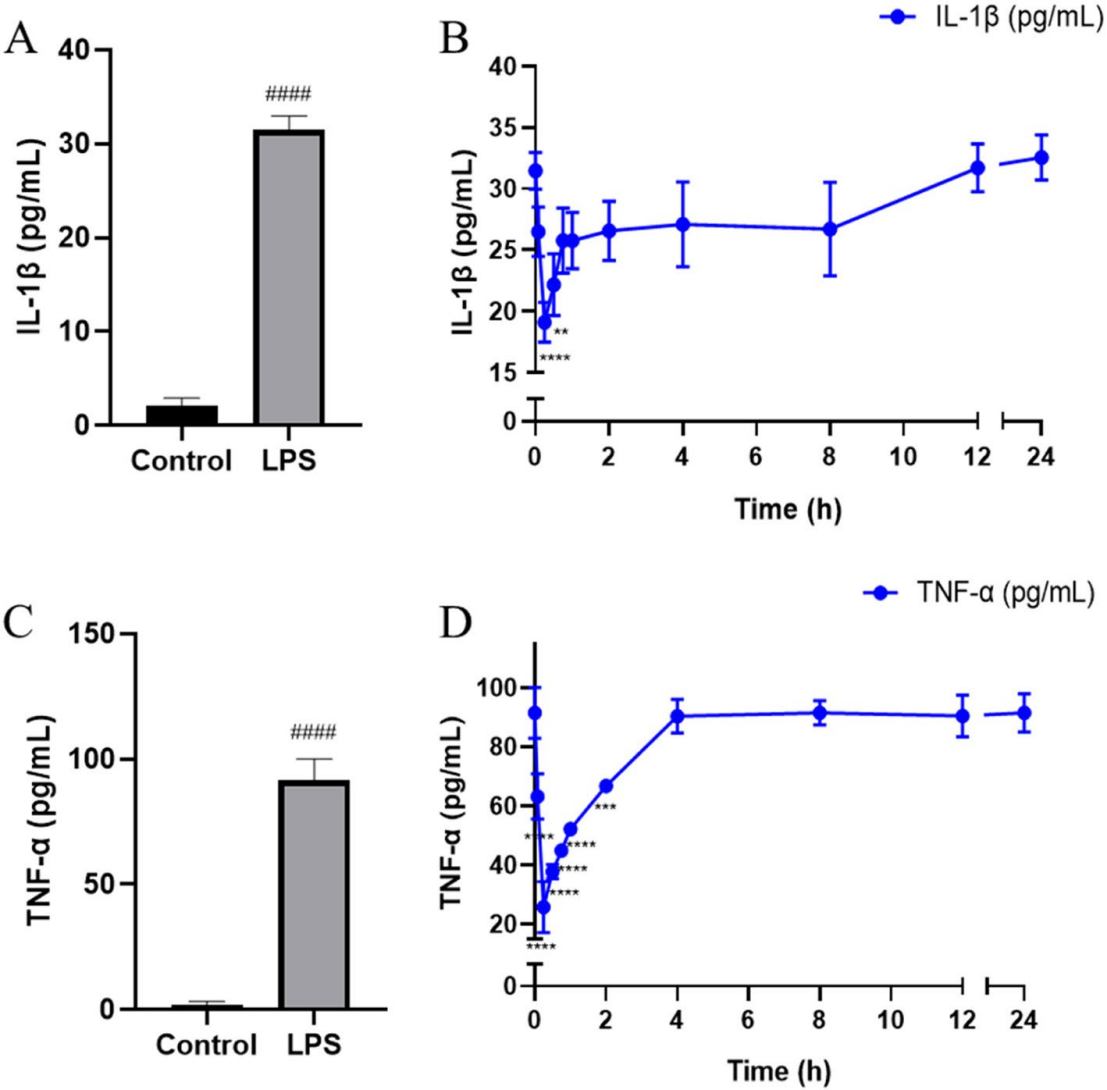
The anti-inflammatory effect of drug-containing plasma on THP-1 cells at different time points. **A** Effect of LPS on the expression of IL-1β. **B** Changes of IL-1β levels after administration. **C** Effect of LPS on the expression of TNF-α. **D** Changes of TNF-α levels after administration. Samples at 0 points were corresponded to blank plasma group. Samples at other time points were corresponded to plasma collected at 0.083, 0.25, 0.5, 0.75, 1, 2, 4, 8, 12, and 24 h, respectively (Samples compared with control group, ####*P* < 0.0001, mean ± SD, *n* = 6. Samples compared with blank plasma group, ****P < 0.0001, ***P < 0.001, **P < 0.01, mean ± SD, *n* = 6)

The inhibitory effect of drug-containing plasma on THP-1 cells at different time points were shown in Fig. 4. Results showed that 0.25 h drug-containing plasma could significantly inhibit the production of IL-1β and TNF-α in cell culture medium, and then the inhibitory effects weakened gradually. Compared with 0 h, IL-1β level was reduced by about 39.31%, and TNF-α was reduced by about 71.86% at 0.25 h. The production of IL-1β remained basically stable at 4–8 h while gradually increased at 8–12 h, and it tended to be smooth at 12 h after administration. Compared with 0.25 h, IL-1β expression was increased by about 66.10%, and TNF-α was increased approximately 2.51-fold at 12 h. There was a phenomenon that the change of efficacy lagged behind the plasma concentration change.

### Pharmacokinetic and pharmacodynamic model fitting

**Fig. 5:**
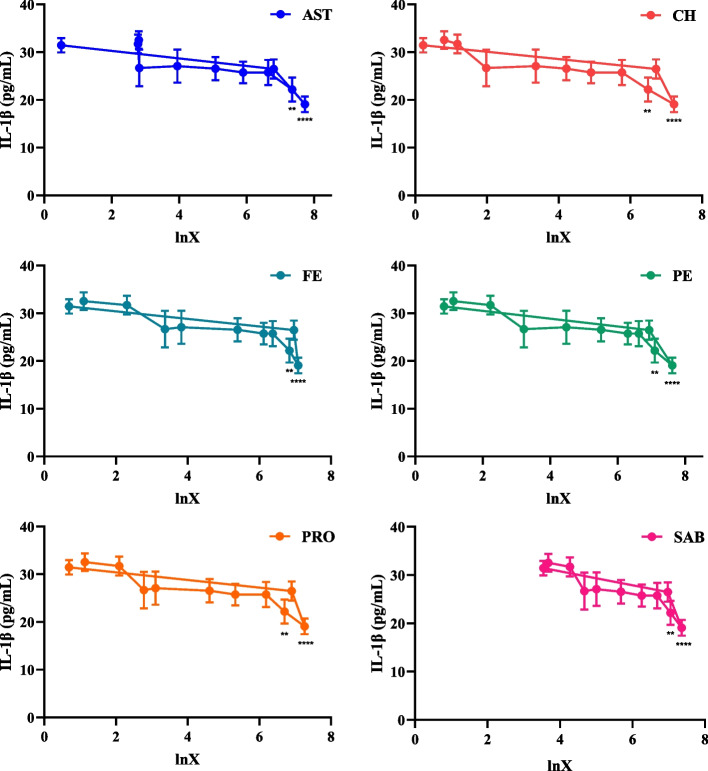
Effect diagram of plasma drug concentration-IL-1β. The abscissa represents the values of *Ln* (plasma drug concentration), and the ordinate represents the changes of IL-1β expression (compared with blank plasma group, ****P < 0.0001, ***P < 0.001, **P < 0.01, *P < 0.05, mean ± SD, *n* = 6)

**Fig. 6:**
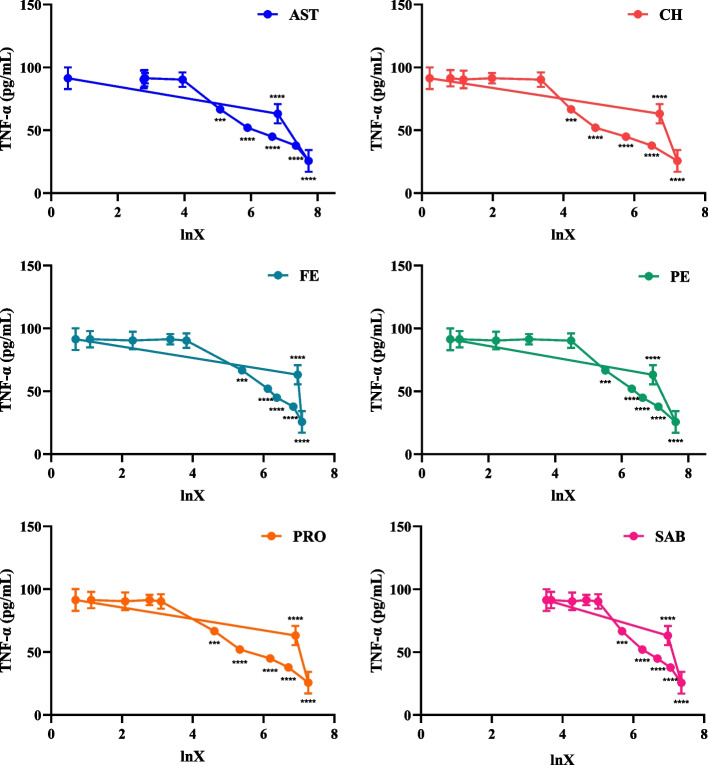
Effect diagram of plasma drug concentration-TNF-α. The abscissa represents the values of *Ln* (plasma drug concentration), and the ordinate represents the changes of TNF-α expression (compared with blank plasma group, ****P < 0.0001, ***P < 0.001, **P < 0.01, *P < 0.05, mean ± SD, *n* = 6)

The PK-PD model fitting results between the plasma concentrations of six compounds and the ability of drug-containing plasma to inhibit the secretion of IL-1β and TNF-α by THP-1 inflammatory cells were shown in Table 6. The results in Figs. 5 and 6 showed that with the increase of drug concentration, the production of IL-1β and TNF-α decreased gradually. When lnX was near 7.4 (0.25 h), the production of IL-1β and TNF-α decreased to a low peak. As the blood concentration of the compounds was decreased, the production of IL-1β and TNF-α showed a trend of gradual increase.

**Table 6:** The fitting equations of six ingredients

ingredients	fitting equation		r	
	**effect fitting of blood concentration—IL-1β**	**effect fitting of blood concentration—TNF-α**	**effect fitting of blood concentration—IL-1β**	**effect fitting of blood concentration—TNF-α**
AST	Y = 33.736—1.448 lnX	Y = 113.618—16.262 lnX	-0.746	-0.900
CH	Y = 32.247—1.386 lnX	Y = 101.980—9.387 lnX	-0.776	-0.891
FE	Y = 33.427—1.446 lnX	Y = 108.880—8.805 lnX	-0.764	-0.867
PE	Y = 33.598—1.428 lnX	Y = 109.393—9.030 lnX	-0.782	-0.858
PRO	Y = 33.031—1.454 lnX	Y = 107.704—8.778 lnX	-0.768	-0.883
SAB	Y = 40.971—2.540 lnX	Y = 158.216—9.637 lnX	-0.781	-0.893

## Discussion

The clinical application of TCM has the characteristics of multi-component, multi-target, and multi-pathway. Researches on its material basis, in vivo processes, pharmacological effects, and their interrelationships have become quite complex. The application of PK-PD model to the study of TCM compound is of great significance to clarify the material basis, the mechanism of action, and the interrelationships [26–28]. Due to the large individual differences in animals and many interference factors, different individuals may show different responses to pharmacodynamic indexes. Besides, limited by detection methods and techniques, some in vivo effect indexes may not accurately reflect the dynamic changes of drugs. With the deepening of biotechnology and molecular level research, the in vitro effect index plays an increasingly important role in this field. Direct action of drugs on isolated tissues, organs, or cells makes the observed pharmacological effect more observable and sensitive [29]. No matter how complex the components of TCM compound are, only the components absorbed and transformed in vivo can exert the efficacy [30]. Accordingly, the pharmaceutical ingredients in plasma represent the active components of TCM compound. Plasma pharmacology can not only effectively eliminate many interference factors in vitro experimental caused by TCM compound and its crude extracts, but also accurately and truly reflect the efficacy and mechanisms of action of TCM compound. Therefore, based on previous network pharmacological study, six absorbable active ingredients (AST, CH, FE, PE, PRO, and SAB) in PYQ were selected as the target ingredients in this study, and PK-PD model was used to study the determination results of anti-inflammatory indexes of PYQ drug-containing plasma in vitro and pharmacokinetic data in rats.

In the present study, an HPLC–MS/MS method was successfully established and validated to determine the six components in PYQ with a good specificity, stability, precision, and accuracy. Meanwhile, a THP-1 inflammatory cell model was induced by LPS, which has been widely used as an in vitro model of human monocytes and macrophages in mechanistic studies of inflammatory diseases. Exposure of THP-1 monocytes to LPS results in an activation of the NF-κB transcription factor, which orchestrates a gene expression program leading to the activation of inflammation, cell proliferation, differentiation, migration, and cell survival, and this activation is mediated by the release of chemokines and cytokines [31]. Cytokines are produced by immune cells and inflammatory cells, which regulate local and systemic immune responses through signal transduction and control the surrounding environment. TNF-α is released in large quantities in inflammatory response, and participates in inflammatory response by promoting the release of other inflammatory cytokines and enhancing the breakdown of inflammatory extracellular proteins, causing histopathological injury in patients with chronic pelvic inflammatory disease [32, 33]. IL-1β is an important inflammatory cytokine secreted by macrophages, which can participate in the occurrence of chronic pelvic inflammation by inducing pro-inflammatory cytokines to accumulate in endothelial cells [33, 34]. If these pro-inflammatory cytokines in pelvic inflammatory disease are decreased, the excessive activation of inflammatory cells can be reduced, and the occurrence and development of PID can be inhibited. Therefore, the levels of inflammatory cytokines including IL-1β and TNF-α in the culture medium of THP-1 inflammatory cells model were taken as the pharmacodynamic index for plasma pharmacology study.

There was a phenomenon that the maximum pharmacodynamics effect was lagged behind the peak value of drug plasma concentration. Subsequently, the blood concentration of chosen target components gradually decreases in 4–12 h and the pharmacological efficacy also disappears. Therefore, these six substances could be the main active substances with in PYQ, and it is speculated that the main active substances could be quickly absorbed into tissues for a prolonged pharmacological effect which still needs further verification.

The results of this study showed that the six components could reach the peak blood concentration within 0.29 h, and there was a potential good correlation between their pharmacokinetics and the pharmacodynamics of PYQ. These results suggest that six compounds may be the main material basis of the PYQ’s therapeutic effect, providing a scientific basis for its clinical application. However, there are some limitations within this study. Firstly, the six major active compounds could not totally reflect all the pharmacodynamic substances of PYQ. Secondly, post-stimulation administration is also a very important aspect of the pharmacological action of PYQ, and further trials will be conducted.

## Conclusions

Based on previous network pharmacological study, six active components (AST, CH, FE, PE, PRO and SAB) in PYQ were selected as the target compounds in this study. Combined with the plasma pharmacological study with THP-1 inflammatory cells model, the PK-PD characteristics of each target compounds were studied. The six components could reach the peak blood concentration within 0.29 h, indicating rapid absorption of the drug. The plasma obtained at 0.25 h point exhibited the best inhibition effect on production of IL-1β and TNF-α in LPS-induced THP-1 cells. There was a good correlation between pharmacokinetics and pharmacodynamics.

## Supplementary Information

**Additional file 1: Figure S1.** The effect of PYQ and its active compounds on the viability of THP-1 cells.

## Data Availability

The datasets used and/or analyzed during the current study available from the corresponding author on reasonable request.

## Abbreviations

PYQ: Penyanqing
TCM: Traditional Chinese medicine
PID: Pelvic inflammatory disease
PK-PD: Pharmacokinetics-pharmacodynamics
LPS: Lipopolysaccharide
THP-1: Human myeloid leukemia mononuclear cells
SAB: Salvianolic acid B
PRO: Protocatechualdehyde
PE: Paeoniflorin
AST: Astilbin
FE: Ferulic acid
CH: Chlorogenic acid
GO: Gene Ontology
KEGG: Kyoto encyclopedia of genes and genomes
HPLC–MS/MS: High performance liquid chromatography-tandem mass spectrometry
TNF-α: Tumor necrosis factor-α
IL-1β: Interleukin-1β
MRTs: Mean residence times
FPR2: Formyl peptide receptor 2
ELISA: Enzyme-linked immunosorbent assay
FBS: Fetal bovine serum
PMA: Phorbol 12-myristate 13-acetate
MRM: Multiple reaction monitoring
IS: Internal standard
QC: Quality control
RSD: Relative standard deviation
RE: Relative error
SD: Standard deviation
LLOQ: Lower limit of quantification
AUC: Area under the curve
t_1/2_: Elimination half-life
*t*_*max*_: Time of maximum concentration
CLz/F: Clearance
Vz/F: Volume of distribution
*C*_*max*_: Maximum concentration
