# Supplementary material for: Pharmacokinetics and pharmacodynamics of bioactive compounds in Penyanqing preparation in THP-1 inflammatory cells induced by Lipopolysaccharide
Source: BMC Complement Med Ther. 2022 Dec 6;22:323. doi: 10.1186/s12906-022-03784-x (PMC9727977; doi:10.1186/s12906-022-03784-x)
Supplement: Supplementary file 1 — Additional file 1: Figure S1. The effect of PYQ and its active compounds on the viability of THP-1 cells. [file 12906_2022_3784_MOESM1_ESM.docx]

Figure S1. The effect of PYQ and its active compounds on the viability of THP-1 cells (compared with the control group, ** *P* < 0.01, **P* < 0.05, *n* = 6)
